# Incarcerated left-sided Amyand’s hernia and synchronous ipsilateral femoral hernia: first case report

**DOI:** 10.1186/s40792-023-01597-9

**Published:** 2023-02-01

**Authors:** Franco A. Corvatta, René M. Palacios Huatuco, Santiago Bertone, José F. Viñas

**Affiliations:** 1 General Surgery Department, Hospital Italiano de Buenos Aires, Tte. Gral. Juan Domingo Perón 4190, Ciudad Autónoma de Buenos Aires, Buenos Aires, Argentina; 2 Plastic Surgery Department, Hospital Italiano de Buenos Aires, University of Buenos Aires School of Medicine, Hospital Italiano de Buenos Aires University Institute, Buenos Aires, Argentina; 3 Microsurgery and Abdominal Wall Reconstruction Section, General Surgery Department, Hospital Italiano de Buenos Aires, Buenos Aires, Argentina

**Keywords:** Inguinal hernia, Femoral hernia, Emergency, Appendix, Mesh repair

## Abstract

**Background:**

The finding of a vermiform appendix within the peritoneal sac of an inguinal hernia is called Amyand’s hernia. The reported incidence of Amyand’s hernia and femoral hernia is 1% and 3.8%, respectively. To our knowledge, no cases have been reported in the literature that associate these two entities. We present the first case of incarcerated left-sided Amyand’s hernia and synchronous ipsilateral femoral hernia found during emergency surgery.

**Case presentation:**

A 72-year-old woman was admitted to the Emergency Department for a complicated left inguinal hernia. An inguinotomy was performed that detected a large direct hernial sac and a synchronous femoral hernia. The opening of the inguinal hernia showed the presence of the cecum and the appendix, both without signs of inflammation. The femoral space was evaluated transinguinally, identifying the larger omentum that had slipped into the femoral canal. The primary closure of the posterior wall defect was performed with the McVay technique due to its large size, and then the hernioplasty was completed with a polypropylene mesh. No postoperative complications were reported.

**Conclusions:**

In the context of an incarcerated Amyand’s hernia, the decision to perform an appendectomy in addition to hernia repair with or without mesh will depend on intraoperative findings.

## Background

The protrusion of the vermiform appendix into an inguinal hernia is called “Amyand’s hernia”. The true incidence of Amyand’s hernia is difficult to establish. Large retrospective studies report rates between 0.14 and 1.3% in inguinal hernia series [1]. Most of them occur on the right side, due to the anatomical position of the appendix. However, the presence of Amyand’s hernia on the left side represents an extremely rare entity, with an incidence of up to 9.5% [1, 2]. On the other hand, an incidence of femoral hernias has been reported in patients who received a preoperative diagnosis of inguinal hernia of up to 13% [3]. We present the first case of incarcerated left-sided Amyand’s hernia and synchronous ipsilateral femoral hernia found during emergency surgery.

## Case presentation

**Fig. 1:**
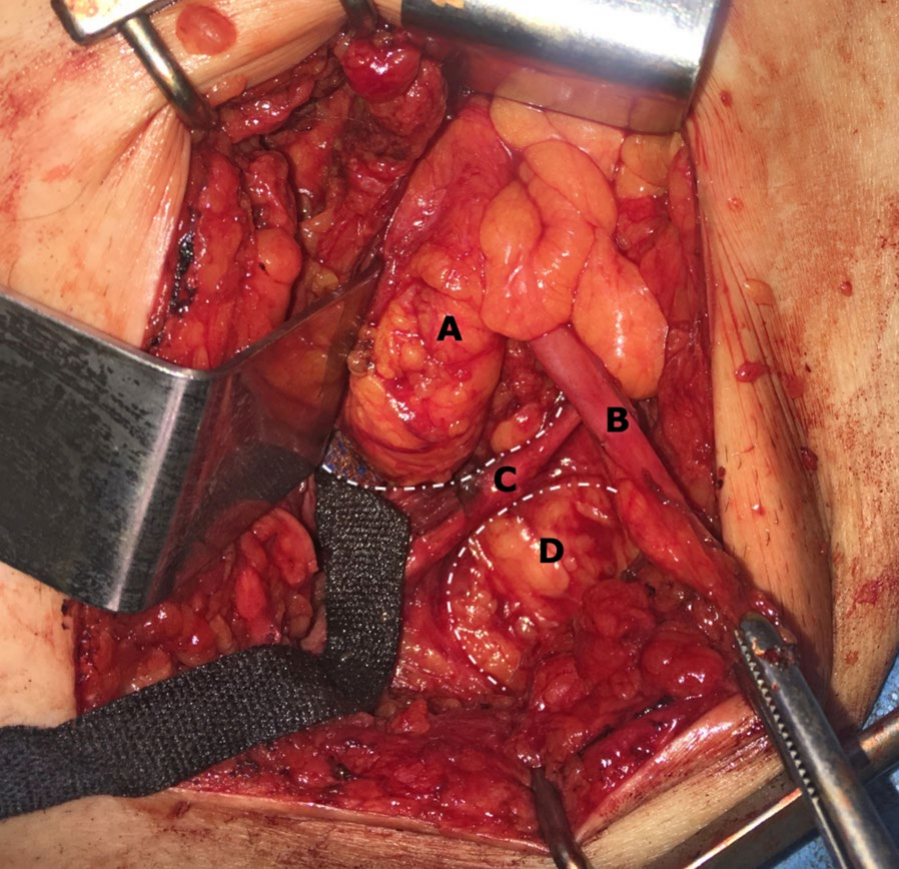
Anterior view of the left inguinal region after dissection and opening of the direct inguinal hernial sac. Gauze was inserted to partially reduce its contents to the abdominal cavity during dissection. **A** cecum, **B** appendix, **C** inguinal ligament, **D** femoral hernia. The dashed dot indicate the boundaries of the inguinal and femoral hernia defects

**Fig. 2:**
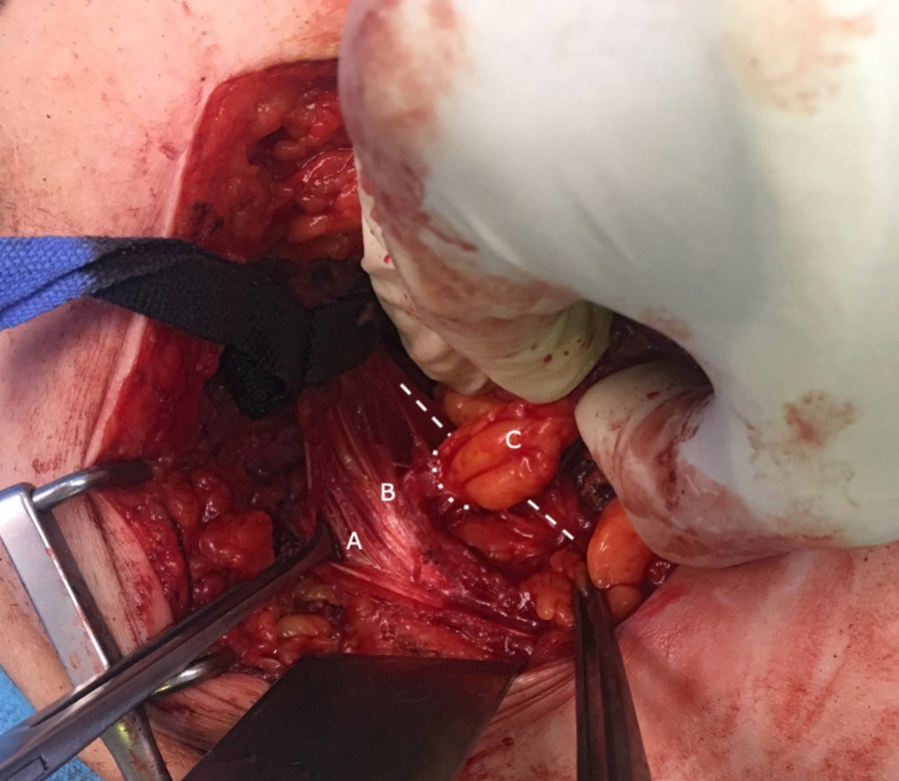
Craniocaudal view of the left inguinal canal and femoral space. **A** Incised external oblique muscle. **B** Inguinal ligament. **C** Greater omentum removed from the femoral canal. The dotted lines indicate the limits of the femoral ring. The colored lines indicate Cooper’s ligament

A 72-year-old woman with a history of chronic obstructive pulmonary disease and arterial hypertension was admitted to the Emergency Department due to abdominal pain and nausea of 24 h evolution. She denied any other symptoms. Upon admission, the patient had a pulse of 112 bpm, blood pressure of 105/85 mmHg, and a temperature of 36.3 °C. On physical examination, her abdomen was distended and tender. An irreducible mass with intense pain on palpation was detected in the left inguinal region. Laboratory tests showed leukocytosis of 11.4 × 10^9^ /L and lactate of 0.7 mmol/L. The metabolic profile and liver function tests were within normal limits. Inguinal ultrasound reported the protrusion of a hollow viscus through a 42 mm fascial continuum. Taking into account the patient's history and the findings of an incarcerated inguinal hernia, emergency surgical treatment was chosen. An anterior approach was performed through an inguinal incision. On examination, a large direct hernial sac protruding from a 6 × 5 cm posterior wall defect and a synchronous ipsilateral femoral hernia were observed. The inguinal hernial sac was opened, showing the presence of the cecum and the appendix (Fig. 1), both without signs of inflammation or ischemia (Losanoff–Basson type 1), so the sac was resected and its contents reintroduced into the abdominal cavity. The femoral space was then evaluated transinguinally, identifying the greater omentum that had slipped into the femoral canal (Fig. 2). Subsequently, a transinguinal dissection of the femoral hernia was performed and its content was reduced. For the reconstructive phase, we consider the existence of a large defect remaining in the posterior wall of the inguinal canal after resection of the hernia sac, associated with the need to repair the found femoral hernia. Given these intraoperative findings, primary closure of the large posterior wall defect and the femoral ring was performed by primary McVay repair. Finally, the hernia repair was completed with the placement of a 15 × 7 cm polypropylene mesh. The procedure was carried out without complications and was discharged on the first postoperative day. No recurrence was detected 12 months after surgery.

## Discussion

To our knowledge, no case has been reported in the literature that associates the presentation of left-sided Amyand’s hernia and synchronous femoral hernia found during emergency hernioplasty. It has been shown in large cohort studies and case series that these two entities have a very low incidence in isolation, so their association could be considered extremely rare.

The pathophysiology of Amyand's hernia is still unclear and its true incidence is difficult to establish. Left-sided Amyand’s hernia has been reported to be usually the result of mobile cecum syndrome and the presence of an excessively long appendix. However, theoretically it can occur in situs inversus or malrotation [2]. Furthermore, in cases of complete appendiceal protrusion into the sac, a portion of the cecum also protrudes [4]. This agrees with our findings and we believe that in our case the pathophysiology of Amyand’s hernia involved the presence of a mobile cecum protruding through the posterior wall of the left inguinal canal.

The clinical picture of Amyand’s hernia is that of an inguinal hernia and is mainly dependent on the inflammatory state of the appendix [5]. It has been reported that up to 83% of cases present with a painful inguinal or inguinoscrotal mass [6], as in the case presented here.

The differential diagnosis is broad and should include an irreducible, incarcerated or strangulated hernia, acute appendicitis, urologic emergencies (testicular torsion, orchiepididymitis, acute hydrocele) and skin complications (inguinal abscess, lymphadenopathy) [1].

Imaging studies are essential for the preoperative diagnosis of Amyand’s hernia, as well as for occult hernias of the inguinal region. Computed tomography (CT) and abdominopelvic ultrasound can demonstrate a tubular structure arising from the cecum and extending into the inguinal sac [7]. However, they are not routine studies when a complicated inguinal hernia is clinically suspected, so in most cases Amyand’s hernia is diagnosed intraoperatively [6]. In the cases of painful, irreducible hernias or suspected incarceration, immediate surgical intervention is clearly indicated, and many surgeons do not perform imaging studies for confirmation [4]. In our report, we did not perform a CT due to the clinical presentation and ultrasound finding of intestinal content stuck in an inguinal hernia, prioritizing emergency surgical treatment.

Regarding the surgical approach, Losanoff and Basson proposed a classification to identify and determine the treatment of Amyand’s hernia, depending on the presence of acute appendicitis and abdominal sepsis [8]. Our case corresponds to a type 1 hernia, defined as an Amyand’s hernia that contains a normal appendix within the hernial sac, for which reduction and repair with mesh is recommended. Although some authors favor routine appendectomy, others do not support it in the absence of inflammation.

Furthermore, even elective appendectomy transforms a clean procedure into a clean contaminated one, slightly increasing the risk of septic complications and mesh infection [9]. We decided not to resect the appendix due to its indemnity, which was reintroduced into the abdominal cavity together with the cecum contained in the hernia sac. Types 2, 3, and 4 involve an inflamed appendix, a perforated appendix, and complicated intra-abdominal pathology, respectively. In these cases, appendectomy and mesh-free hernia repair are recommended.

Recommendations for the treatment of inguinal hernias include mesh and tension-free repair [5]. Furthermore, in the case of direct hernias, the primary closure of the direct defect should be considered once the hernia contents have been reduced [10]. In our case, we decided to perform primary closure of the direct defect with a repair of the posterior wall of the inguinal canal with the McVay technique, due to the large extension of the remaining defect and the concomitant need to repair the femoral ring. As there was no evidence of a contaminated field, we completed the hernia repair by placing of a polypropylene mesh, placed above the primary repair. An alternative for reconstruction would have been an open preperitoneal anterior approach, but this was ruled out due to the large extent of the remaining posterior wall defect after the section of the hernia sac.

Compared to inguinal hernias, femoral hernias are rare and according to different series and cohort studies, they represent 2–13% of inguinal hernia repairs [3, 11]. Unlike inguinal hernias, femoral hernias are more likely to require urgent repair and are associated with a higher rate of complications and morbidity [12].

Given the low incidence of these two entities, systematic reviews of case reports and case series will likely remain the best available level of evidence for the foreseeable future.

## Conclusions

This is the first reported case of an incarcerated left-sided Amyand’s hernia with a synchronous ipsilateral femoral hernia found during an emergency hernioplasty. The decision to perform an appendectomy and hernia repair with or without mesh placement will depend on the intraoperative findings. In addition, open primary tissue approximation nonmesh repairs, such as the McVay technique, are valid alternatives to herniorrhaphy, especially if infection is found.

## Data Availability

The data are not available for public access because of patient privacy concerns, but are available from the corresponding author on reasonable request.

## Abbreviation

CT: Computed tomography
